# Relationship between Receipt of a Social Protection Grant for a Child and Second Pregnancy Rates among South African Women: A Cohort Study

**DOI:** 10.1371/journal.pone.0137352

**Published:** 2015-09-23

**Authors:** Molly Rosenberg, Audrey Pettifor, Nadia Nguyen, Daniel Westreich, Jacob Bor, Till Bärnighausen, Paul Mee, Rhian Twine, Stephen Tollman, Kathleen Kahn

**Affiliations:** 1 Center for Population and Development Studies, Harvard University, Cambridge, United States of America; 2 Department of Epidemiology, University of North Carolina-Chapel Hill, Chapel Hill, United States of America; 3 MRC/Wits Rural Public Health and Health Transitions Research Unit (Agincourt), School of Public Health, Faculty of Health Sciences, University of the Witwatersrand, Johannesburg, South Africa; 4 Carolina Population Center, University of North Carolina-Chapel Hill, Chapel Hill, United States of America; 5 Department of Global Health, Boston University, Boston, United States of America; 6 Department of Global Health and Population, Harvard School of Public Health, Harvard University, Boston, United States of America; 7 Wellcome Trust Africa Centre for Health and Population Studies, Mtubatuba, KwaZulu-Natal, South Africa; 8 Department of Global Health and Development, Faculty of Public Health and Policy, London School of Hygiene and Tropical Medicine, London, United Kingdom; 9 Umeå Centre for Global Health Research, Division of Epidemiology and Global Health, Department of Public Health and Clinical Medicine, Umeå University, Umeå, Sweden; 10 INDEPTH Network, Accra, Ghana; University Hospital Basel, SWITZERLAND

## Abstract

**Background:**

Social protection programs issuing cash grants to caregivers of young children may influence fertility. Grant-related income could foster economic independence and/or increase access to job prospects, education, and health services, resulting in lower pregnancy rates. In the other direction, these programs may motivate family expansion in order to receive larger grants. Here, we estimate the net effect of these countervailing mechanisms among rural South African women.

**Methods:**

We constructed a retrospective cohort of 4845 women who first became eligible for the Child Support Grant with the birth of their first child between 1998 and 2008, with data originally collected by the Agincourt Health and Socio-Demographic Surveillance System in Mpumalanga province, South Africa. We fit Cox regression models to estimate the hazard of second pregnancy in women who reported grant receipt after birth of first child, relative to non-recipients. As a secondary analysis to explore the potential for grant loss to incentivize second pregnancy, we exploited a natural experiment created by a 2003 expansion of the program’s age eligibility criterion from age seven to nine. We compared second pregnancy rates between (i) women with children age seven or eight in 2002 (recently aged out of grant eligibility) to (ii) women with children age seven or eight in 2003 (remained grant-eligible).

**Results:**

The adjusted hazard ratio for the association between grant exposure and second pregnancy was 0.66 (95% CI: 0.58, 0.75). Women with first children who aged out of grant eligibility in 2002 had similar second pregnancy rates to women with first children who remained grant-eligible in 2003 [IRR (95% CI): 0.9 (0.5, 1.4)].

**Conclusions:**

Across both primary and secondary analyses, we found no evidence that the Child Support Grant incentivizes pregnancy. In harmony with South African population policy, receipt of the Child Support Grant may result in longer spacing between pregnancies.

## Introduction

Cash transfers are widely used in social protection programs for poverty alleviation. Many such programs focus on providing support for children by providing monthly cash transfers to families to help them meet basic needs. Typically, these programs increase support incrementally as number of children increases. Critics of such programs have argued that this condition can create perverse incentives for women to have more children to receive a larger monthly payment.[1–5]. Although some qualitative evidence suggests this ‘incentive effect’ may exist,[6] few empirical studies substantiate such concern.[7, 8]

It is also plausible that social protection programs may not influence fertility at all,[8–10] or may be associated with decreased fertility.[11] A lack of association could be explained if the amount of money provided by the program is simply not large enough to incentivize dramatic fertility-related behavior change. Alternatively, an ‘income effect’ may explain lower pregnancy rates if the extra income provided by the program facilitates access to health services (including family planning resources), improves job prospects, or increases female economic independence relative to her partner.[12–14] Thus, the direction of the relationship between social protection programs and fertility is theoretically ambiguous as the relative contributions of the incentive and income effects are unknown.

The South African Child Support Grant (CSG) has not been found to be associated with increases in teenage pregnancy in previous studies.[15–20] However, much of this work relies on ecological data to draw conclusions about the impact of the CSG on individuals.[15–18] Moreover, all prior studies have focused exclusively on teenage fertility while the question of whether the CSG influences fertility decisions is relevant to all women of reproductive age.

In this study, we aim to establish for the first time the net fertility effects of the CSG using longitudinal, individual-level data. Understanding how, if at all, the CSG may influence fertility is critical to establish whether or not the grant unintentionally incentivizes pregnancy, and to better understand how economic inputs may influence fertility-related decisions.

## Methods

To explore the relationship between the Child Support Grant (CSG) and fertility, we conducted two distinct, but related, analyses. In our primary analysis, we used observational data from the Agincourt health and socio-demographic surveillance site to examine how receipt of the CSG after the birth of first child was associated with timing of second pregnancy. In our secondary analysis, we conducted a natural experiment using data from two socio-demographic surveillance sites (Agincourt and Africa Centre) to examine the influence of loss of the CSG due to age ineligibility on second pregnancy rates.

### Child Support Grant

The CSG was established in 1998, and, after initial low-uptake,[21] is now South Africa’s largest social protection program, with more than 10 million child beneficiaries.[22, 23] To be eligible in 1998, caregivers needed to care for a child under seven; belong to a household living in poverty, ascertained by a means test; and have proof of their and their child’s South African citizenship.[23, 24] Since its introduction, the grant payment has increased from 100 to 330 Rand (from about US$8 to US$27) per month per child and age eligibility has been repeatedly broadened over time. The program was expanded in April 2003 to include children up to age nine; in April 2004 up to age 11; in April 2005 up to age 14; in April 2009 up to age 15; and in April 2010 up to age 18.[22]

### Study population

For the primary analysis, we assembled a cohort of women from a health and socio-demographic surveillance site (HDSS) located in the Agincourt sub-district of Bushbuckridge in rural Mpumalanga Province, South Africa. The Agincourt HDSS, run by the Medical Research Council/Wits University Rural Public Health and Health Transitions Research Unit, is a longitudinal, population-based, full community cohort study that has continually monitored all vital events of all people living in the study area since 1992. The HDSS now covers over 110,000 people living in 21,000 households across 31 villages. The household census, updated annually, collects information on all births, deaths, and in- and out-migrations. Other important individual and household-level information is collected on a regular, but less frequent, basis. Details of the data collection methods have been described previously.[25, 26]

We also performed a secondary analysis pooling Agincourt HDSS data with data from the Africa Centre Demographic Information System (ACDIS). Since 2000, ACDIS has collected longitudinal, population-based health and demographic data on a full community cohort located in the uMkhanyakude district of northern KwaZulu-Natal. Currently, ACDIS covers about 110,000 individuals in over 11,000 households.[27, 28] In comparison, the Agincourt HDSS and ACDIS both collect longitudinal demographic and health data on every person living within their respective communities, and they both cover poor, rural communities of similar size with respect to both population and area. In contrast, the Agincourt HDSS covers a population that is slightly wealthier than that covered by ACDIS, and the Agincourt population is subdivided into distinct villages while the ACDIS population is not grouped in this way.[29]

Community, household, and individual consent have been obtained for all Agincourt HDSS research since its inception. Informed verbal consent is obtained at each household follow-up visit and participant consent records are retained on each household roster. Ethics approval for Agincourt HDSS research, including the consent procedures, was obtained from the University of the Witwatersrand’s Committee for Research on Human Subjects (updated # M110138; original # M960720) and the Mpumalanga Province Health Research and Ethics Committee. Ethics approval for ACDIS data collection is obtained annually from the University of KwaZulu-Natal’s Biological Research Ethics Committee. Ethics approval for this analysis was obtained from the Office of Human Research Ethics at the University of North Carolina—Chapel Hill (#11–1605) and the Harvard Human Research Protection Program (#14–3909).

### Key measures

As the CSG can only be received after a woman has had her first child, we examined the individual impact of grant receipt on *second pregnancy*. During each Agincourt HDSS and ACDIS census update, a household respondent identifies any currently or recently pregnant women living in the household. All recently pregnant women are then followed-up to collect a range of pregnancy-specific information, including the date of delivery. We back-calculated the estimated conception date of each second pregnancy by subtracting 40 weeks from the delivery date of the second child. Time to second pregnancy was defined as the interval, in days, between the delivery of the first child and the estimated conception date of the second child. Women who did not go on to have a second child during follow-up were censored at the end of the follow-up period.

Our primary exposure of interest was *receipt of the CSG*. The HDSS collected data regarding the CSG in 2002, 2005, and 2008. Respondents reported whether or not the grant was received and the date on which it was first received. We partitioned the dataset so that participants who became exposed contributed unexposed person-time up until CSG receipt and exposed person-time thereafter. In the secondary analysis, our exposure of interest was *loss of CSG eligibility*. To define loss of CSG, we exploited a natural experiment created by a 2003 age expansion of the grant. In April 2003, the age cut-off for CSG eligibility was extended from age seven to age nine. Thus, women with children age seven or eight in the year prior to the age expansion recently lost eligibility for the grant. Women with children age seven or eight in the year after the age expansion maintained eligibility for the grant.

We used a directed acyclic graph to identify a minimally sufficient adjustment set of potential confounders of the relationship between CSG receipt and time to second pregnancy. Specifically, we adjusted for: *age*, at the time of first birth; *household wealth*, measured with an index of household assets;[30] *education*, measured as years of educational attainment; *marital status*, categorized as never married, married, or divorced/separated/widowed; *former refugee status*, measured by a variable that identifies individuals who were ever refugees of Mozambican descent; and *calendar year*, measured as the year of birth of first child.

### Statistical analyses

We examined the relationship between CSG receipt and second pregnancy using Cox regression models to estimate the hazard ratio (HR) for CSG recipients compared to non-recipients. Women who lived in the study area between January 1, 1998 and December 31, 2008 began contributing person-time after the birth of their first child. Women were censored at the estimated conception date of their second child or at the end of follow-up (whichever came first). Women who died or moved out of the study area before their second pregnancy were censored at the time of death or move. Women who received the grant for a non-biological child prior to the birth of their first biological child were excluded from the cohort.

We adjusted our models for the potential confounding effects of age, household wealth, education, marital status, former refugee status, and calendar year of birth of first child. Continuous covariates were coded flexibly with restricted cubic splines. All time-varying covariates were updated each time new data were provided. To address concerns that receipt of the CSG could influence subsequent time-varying covariates and lead to biased estimates of the total effect, we also estimated a marginal structural model (MSM) using inverse probability of treatment weights.[31] To assess whether the observed association differed by age, we compared HRs from models among women who were under age 21 and women who were age 21 or older at the time of the birth of their first child.

We also calculated weighted extended Kaplan-Meier (KM) curves [32, 33], plotting the KM estimator-derived cumulative incidence of second pregnancy against time since birth of first child, by CSG status. To construct the curves, we used stabilized inverse probability of treatment weights to control for age, education, household wealth, refugee status, and calendar year of first birth, and then calculated the KM estimators in the re-weighted population. Absolute differences in time to second pregnancy by exposure status were calculated at 25^th^ and 50^th^ percentiles, using a bootstrapping method to estimate 95% confidence intervals.

**Fig 1:**
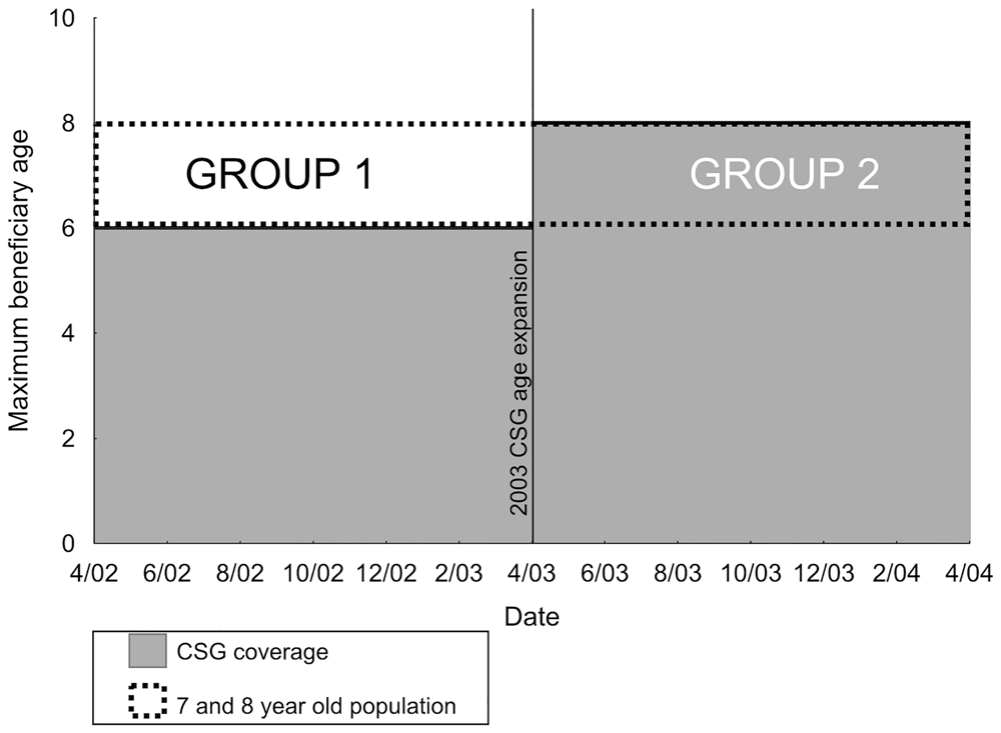
Schematic of the April 2003 Child Support Grant age eligibility expansion, shifting the maximum age of a grant-eligible child from seven to nine. CSG = Child Support Grant. Group 1 = 7 and 8 year olds in the year prior to the April 2003 age expansion who recently lost age-eligibility for the Child Support Grant. Group 2 = 7 and 8 year olds in the year after the April 2003 age expansion who maintain their age-eligibility under the new rule. We compared second pregnancy rates among women with first children in Group 1 to women with first children in Group 2.

To explicitly test for evidence of an incentive effect, as a secondary analysis, we exploited a natural experiment created by the April 2003 expansion of child age eligibility for the CSG. We reasoned that if the CSG incentivized pregnancy, women with first children who aged out of CSG-eligibility would have increased second pregnancy rates in order to regain CSG eligibility with a new child. Conversely, women with first children who remained age-eligible would not have the same incentive to have a second child because they still received the CSG benefits for their first child. The 2004 age-expansion abruptly shifted the maximum age of a grant-eligible child from seven to nine years old (Fig 1). Thus, we compared second pregnancy rates between two groups: 1. Among women with a first child aged seven or eight between April 2002—March 2003 (children who recently became CSG-ineligible), and 2. Among women with a first child aged seven or eight between April 2003—March 2004 (children who remained CSG-eligible), using Poisson regression models. We compared estimates using data from Agincourt HDSS and ACDIS; estimates of similar magnitude were pooled across sites. All analyses were performed using SAS v.9.2 [34].

## Results

**Fig 2:**
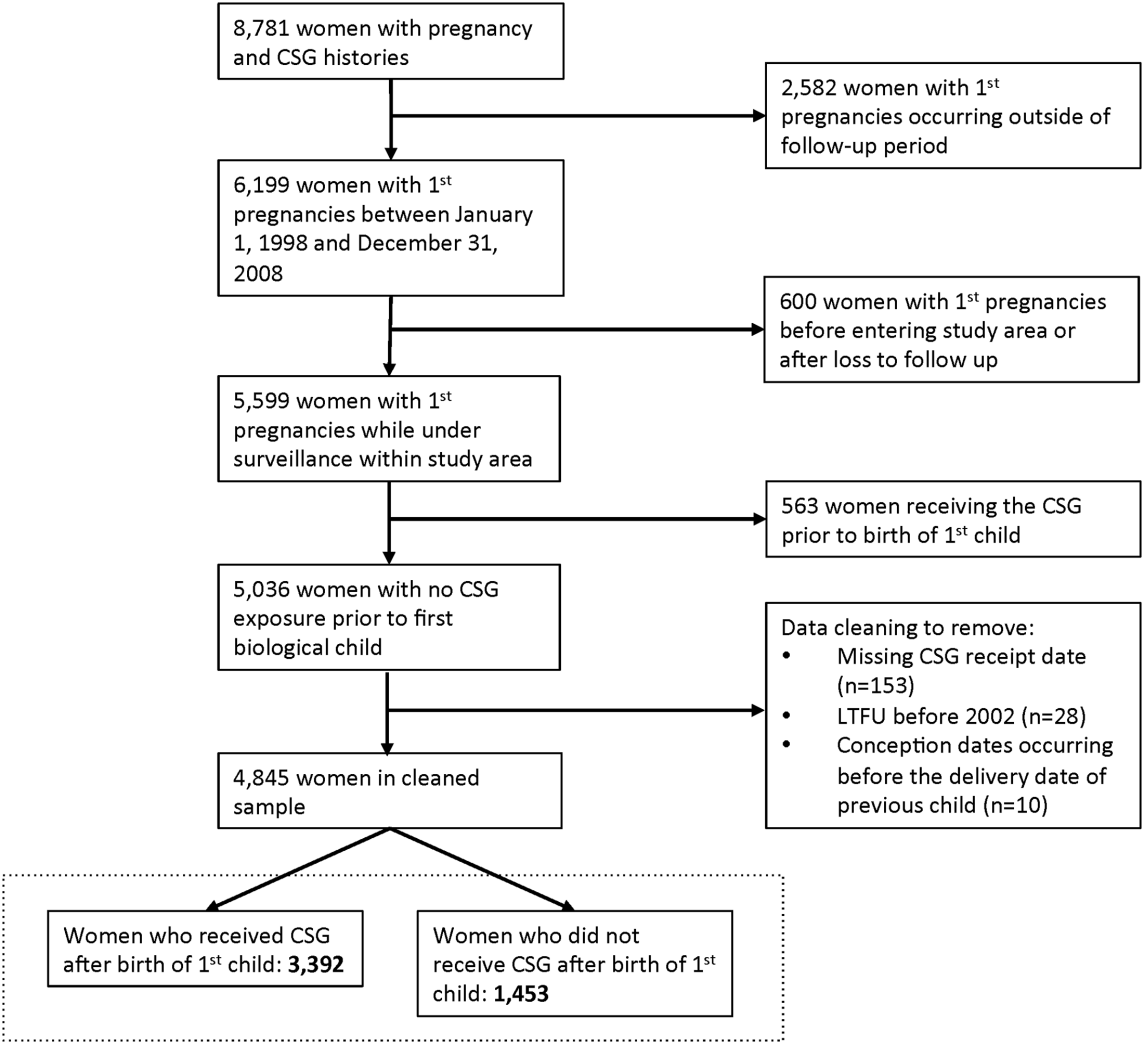
Flowchart of cohort construction of 4,845 women in Agincourt, South Africa, 1998–2008. CSG = Child Support Grant. LTFU = loss to follow-up.

Overall, 8,781 women were observed with CSG and pregnancy histories (Fig 2). After removing: (i) women with first pregnancies occurring outside of the follow-up calendar period (n = 2,582); (ii) women who lived outside of the study area during their first pregnancies (n = 600); and (iii) women who reported CSG receipt for a non-biological child (n = 563); and after data cleaning to remove those with missing or unreliable data (n = 191), the final cohort had a sample size of 4,845 women.

Overall, 70% of women reported CSG receipt after the birth of their first child. There were significant differences in covariate distribution between CSG recipients and non-recipients (Table 1). Women who received the CSG were slightly older at baseline, lived in slightly wealthier households, were more likely to have some formal education, were more likely to be married, and were less likely to be former Mozambican refugees. Also, the median year of first birth was considerably later among CSG recipients (2004) compared to non-recipients (2001), likely reflecting the slow uptake of the program when it was first introduced.[21] A third of all women in the total cohort (33%) went on to have a second child during follow-up. We analyzed a total of 18,840 person-years with an average follow-up time of 3.9 years.

**Table 1:** Distribution of covariates at time of first pregnancy, by CSG receipt status, in a cohort of 4845 women in Agincourt, South Africa, 1998–2008.

	Total (N = 4845)		CSG (N = 3392)		No CSG (N = 1453)		
*Categorical variables*	*N*	*%*	*N*	*%*	*N*	*%*	*p* ^1^
**Education**							
None	337	7.4	207	6.5	130	9.4	<0.0001
Some primary schooling	638	13.9	405	12.7	233	16.8	
Secondary school graduate	2726	59.5	1952	61.1	774	55.7	
Post-secondary schooling	772	16.8	557	17.4	215	15.5	
Missing	n = 260		n = 196		n = 64		
**Marital status**							
Never married	3202	73.4	2187	72.4	1015	75.8	0.03
Married	710	16.3	520	17.2	190	14.2	
Divorced/separated/widowed	448	10.3	313	10.4	135	10.1	
Missing	n = 485		n = 372		n = 113		
**Former Mozambican refugee**							
Yes	1428	29.5	937	27.6	491	33.8	<0.0001
No	3417	70.5	2455	72.4	962	66.2	
Missing	n = 0		n = 0		n = 0		
*Continuous variables*	*Median*	*IQR*	*Median*	*IQR*	*Median*	*IQR*	
**Age**	22	18–27	22	19–27	21	18–27	0.01
Missing	n = 0		n = 0		n = 0		
**Household wealth** ^2^	2.26	1.92–2.55	2.27	1.94–2.55	2.23	1.88–2.51	0.02
Missing	n = 256		n = 194		n = 62		
**Calendar year**	2003	2000–2005	2004	2001–2006	2001	1999–2004	<0.0001
Missing	n = 0		n = 0		n = 0		

^1^For categorical variables, p-value is for a chi-square test; for continuous variables, p-value is for a Wilcoxon rank sum test

^2^Household wealth measured as a composite index based on household assets

CSG = Child Support Grant; IQR = Inter-Quartile Range; CI = Confidence Interval; MSM = Marginal Structural Model

Receipt of the CSG after birth of first child appeared protective against second pregnancy (Table 2). In the unadjusted model among the full cohort, the HR for second pregnancy among CSG recipients compared to non-recipients was 0.72 (95% confidence interval [CI]: 0.65, 0.79). Adjustment for age, education, household wealth, refugee status, and calendar year of first birth did not markedly affect the observed association (aHR: 0.66; 95% CI: 0.58, 0.75), and the results from the MSM were of similar magnitude and precision (HR: 0.65; 95% CI: 0.57, 0.74). We observed a protective association of similar magnitude in both younger (aHR: 0.60; 95% CI: 0.50, 0.71) and older (aHR: 0.72; 95% CI: 0.61, 0.86) women. Results from a likelihood ratio test indicated that the addition of an interaction term between the CSG exposure and the dichotomous indicator for younger versus older maternal age did not significantly improve model fit (chi-square statistic: 0.50, p = 0.8).

**Table 2:** Results from Cox regression models comparing the hazard of second pregnancy among Child Support Grant recipients relative to non-recipients, in a cohort of 4845 women in Agincourt, South Africa, 1998–2008.

Model	HR	95% CI	p-value
**Full cohort** (n = 4845)			
Unadjusted	0.72	0.65, 0.79	<0.0001
Adjusted^1^	0.66	0.58, 0.75	<0.0001
MSM	0.65	0.58, 0.75	<0.0001
**Under 21 years** ^2^ (n = 2110)			
Unadjusted	0.70	0.60, 0.82	<0.0001
Adjusted^1^	0.60	0.50, 0.72	<0.0001
**21 years or older** ^2^ (n = 2735)			
Unadjusted	0.73	0.64, 0.84	<0.0001
Adjusted^1^	0.72	0.61, 0.86	<0.0001

^1^Adjusted for age (coded with a restricted cubic spline with five knots), education (coded, in years, with a restricted cubic spline with five knots), former refugee status (coded as a dichotomous variable), household wealth (coded with a restricted cubic spline with five knots), and calendar year of birth of first child (coded with a restricted cubic spline with five knots)

^2^Age at birth of first child

HR = Hazard Ratio; CI = Confidence Interval; MSM = Marginal Structural Model

**Fig 3:**
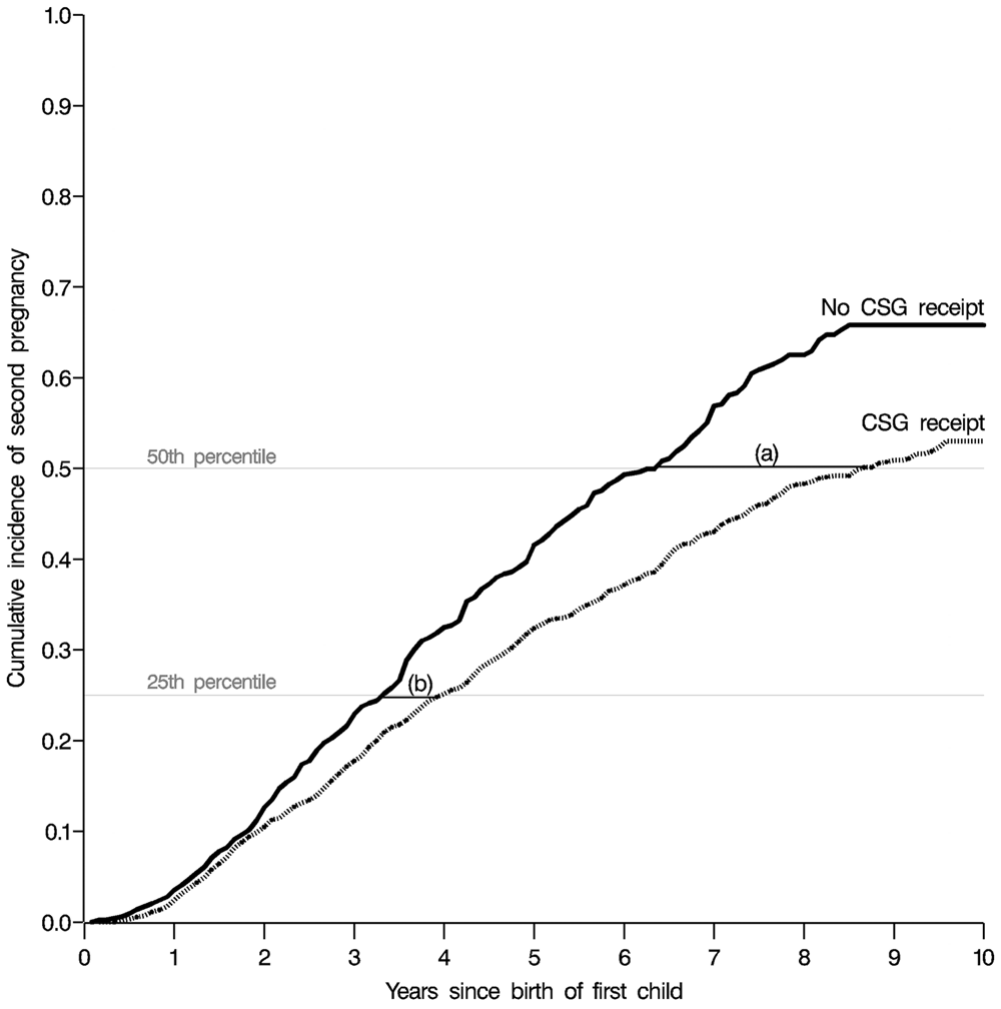
Weighted^1^ extended Kaplan Meier-type cumulative incidence curves for time to second pregnancy among 4,845 women in Agincourt, South Africa, 1998–2008, by exposure to Child Support Grant (CSG). (a) Absolute difference in median time to second pregnancy between CSG recipients and non-recipients: 30 months (95% CI: 12, 42 months). (b) Absolute difference in time to second pregnancy at 25^th^ percentile between CSG recipients and non-recipients: 8 months (95% CI: 2, 14 months). ^1^Weighted using stabilized inverse probability of treatment weights to control for age (coded with a restricted cubic spline with five knots), education (coded, in years, with a restricted cubic spline with five knots), former refugee status (coded as a dichotomous variable), household wealth (coded with a restricted cubic spline with five knots), and calendar year of birth of first child (coded with a restricted cubic spline with five knots)

The weighted KM curve supports the findings from the Cox models (Fig 3). Time to second pregnancy was significantly longer among CSG recipients compared to non-recipients at both the 25^th^ [absolute difference (months): 8; 95% CI: 2,14] and 50^th^ percentiles [absolute difference (months): 30; 95% CI: 12, 42].

Finally, loss of CSG for first biological child was not associated with second pregnancy incidence (Table 3). Women with children who were seven or eight years old between April 2002 and March 2003 (recently became age-ineligible for the CSG) had similar second pregnancy rates as women with children who were seven or eight years old between April 2003 and March 2004 (remained age-eligible for the CSG under a new expansion). This lack of association was observed in both the Agincourt HDSS sample (IRR 0.9; 95% CI: 0.5, 1.4), and in the ACDIS sample (IRR: 0.9; 95% CI: 0.6, 1.4). Given the similarity of these two point estimates, we gained precision by pooling the data across both sites (IRR: 0.9; 95% CI: 0.6, 1.2).

**Table 3:** Second pregnancy rates among those with first children age 7–8 in April 2002-March 2003, compared to April 2003-March 2004, across two demographic surveillance sites in rural South Africa.

	Pregnancies	PY	Rate/100 PY	IRR (95% CI)
*AHDSS*				
Apr. 02 –Mar. 03^1^	32	590	5.43	0.9 (0.5, 1.4)
Apr. 03 –Mar. 04^2^	34	531	6.41	1
*ACDIS*				
Apr. 02 –Mar. 03^1^	39	501	7.79	0.9 (0.6, 1.4)
Apr. 03 –Mar. 04^2^	48	578	8.31	1
*Pooled*				
Apr. 02 –Mar. 03^1^	71	1090	6.51	0.9 (0.6, 1.2)
Apr. 03 –Mar. 04^2^	82	1108	7.40	1

^1^Seven and eight year olds not age-eligible for the Child Support Grant at this time

^2^Seven and eight year olds age-eligible for the Child Support Grant at this time

PY = Person Years; IRR = Incidence Rate Ratio; CI = Confidence Interval; AHDSS = Agincourt Health and socio-Demographic Surveillance System; ACDIS = Africa Centre Demographic Information System

## Discussion

In this paper, we present evidence that receipt of the Child Support Grant was significantly associated with lower second pregnancy rates. In the context of South Africa, where fertility remains above replacement level among many socioeconomically disadvantaged groups,[35] official population policy expresses objectives to both reduce unplanned and unwanted pregnancies and to reduce poverty and socioeconomic disparities.[36] Our results suggest that the Child Support Grant may, in fact, meet both of these objectives. Much concern about the potential for social welfare grants to incentivize pregnancy has been focused on teenagers. However, in the age-stratified analysis, we found that the protective association of the grant with second pregnancy rates existed in both younger and older women. Further, we found no evidence that women with children who became grant-ineligible had different second pregnancy rates than women whose children remained grant-eligible. None of our findings support the notion that the Child Support Grant leads to higher fertility.

There are several plausible mechanisms to explain how CSG receipt may lead to decreased pregnancy rates through an income effect. Economic independence facilitated by the grant may shift relationship power dynamics, leading to improved agency with respect to sexual and reproductive health decision-making, or decreasing the need for transactional-sex based relationships.[14] Similarly, grant-associated improvements in education and job prospects may increase the opportunity costs of pregnancy and motherhood, while improved access to health services may facilitate higher contraception use.[12, 13] Although our findings lend support to the notion that an income effect is operating to suppress fertility, we cannot rule out that an incentive effect is also operating to increase fertility. However, given that we observe a net effect of decreased fertility, if the incentive effect exists, it likely operates at a smaller magnitude relative to the income effect. Future qualitative studies could provide further insight into the pathways through which this grant may be influencing fertility decisions.

The use of long-term, longitudinal, population-based HDSS data allowed us to draw stronger inferences over prior work. We conducted an individual-level analysis in a full community cohort, as opposed to the ecological analyses in many prior studies,[15–18] which allowed us to confidently interpret our findings at the individual-, as opposed to population-, level, and reduced the possibility for selection bias. Our findings are in line with the protective association observed among teenage recipients [19] and teenage beneficiaries [20] in the two studies using data at the individual-level. Also, we utilized a long follow-up period to estimate the association between the CSG and fertility in the first eleven years since the program was established. The length of follow-up is an important advantage to this study as changes to family structure occur over long time periods. This is especially true in South Africa where long spacing between first and second children is common, consistent with our findings.[37–40] Finally, our secondary results are strengthened by comparing, and eventually pooling, results across both demographic surveillance sites (Agincourt HDSS and ACDIS). The similarity of the point estimates across sites lends credence to the external validity of our results in rural South Africa.

This study examines some, but not all, of the ways the CSG could influence fertility. Specifically, we focus on receipt (and loss) of the grant as the exposure of interest. We assume grant receipt is related to both (i) awareness of grant and (ii) real-life experience with how much the costs associated with having children are (or are not) offset by the grant. Grant receipt is also clearly associated with increased income, which, as discussed above, is a plausible mechanism for the protective association we observed. The comparisons we make between grant recipients and non-recipients allow us to take advantage of the detailed, individual-level grant exposure data in the Agincourt HDSS. However, it restricts us to examining the effects of the grant on second order pregnancies or higher, as a first child is necessary to receive the grant. Whether or not the program incentivizes first pregnancies is not explored in this paper. Future studies, perhaps exploiting differences in the accessibility of the program in early years, could be conducted to examine this important question.

Importantly, exposure to the grant was not randomly assigned in our study population. In our primary analysis, those who received the grant after the birth of their first child had significantly different covariate distributions than those who did not receive the grant. To account for the influence of potential confounders, we adjusted our models to close all identified confounding paths, updating the covariates whenever new information was available, and coding them flexibly with restricted cubic splines. Our point estimates were essentially identical before and after covariate adjustment. However, it is possible that the women who received the grant were different in other unmeasured ways than women who did not receive the grant (i.e. ability to navigate barriers to CSG access), which could confound our estimates.

As the evidence continues to come in about the benefits provided by social protection programs in economic, health, and education realms [12], it is important to also explore any unplanned outcomes the programs may have, including changes in fertility. This study provides evidence that receipt of the Child Support Grant in rural South Africa is associated with significant delays in time to second pregnancy. Researchers and policymakers alike should further consider the potential for social protection grants to reduce unwanted and unplanned fertility.
